# Fractures incidence and its association on mortality in multiple myeloma patients: a nationwide cohort study (CAREMM-2105 study)

**DOI:** 10.1038/s41598-025-09811-4

**Published:** 2025-07-27

**Authors:** Jeonghoon Ha, Suein Choi, Sung-Soo Park, Seulji Moon, Jinseon Han, Jeongyoon Lee, Ki-Hyun Baek, Seunghoon Han, Chang-Ki Min

**Affiliations:** 1 Division of Endocrinology and Metabolism, Department of Internal Medicine, Seoul St. Mary’s Hospital, College of Medicine, The Catholic University of Korea, Seoul, Republic of Korea; 2 Division of Endocrinology, Stanford University School of Medicine, Stanford, CA USA; 3 Department of Pharmacology, College of Medicine, The Catholic University of Korea, Seoul, Republic of Korea; 4 Division of Data Science, PIPET, College of Medicine , The Catholic University of Korea, Seoul, Republic of Korea; 5 Division of Hematology, Seoul St. Mary’s Hematology Hospital, College of Medicine, The Catholic University of Korea, Seoul, Republic of Korea; 6 Catholic Research Network for Multiple Myeloma, Seoul, Republic of Korea; 7 Division of Endocrinology and Metabolism, Department of Internal Medicine, Yeouido St. Mary’s Hospital, College of Medicine, The Catholic University of Korea, Seoul, Republic of Korea

**Keywords:** Multiple myeloma, Fracture, Mortality, Bone health, Quality of life, Cancer epidemiology, Cancer, Endocrinology, Oncology

## Abstract

Multiple myeloma (MM) is known to compromise bone integrity, leading to an increased risk of fractures, which compromise the quality of life and increase mortality rates. This study investigated the incidence of fractures in MM patients and explored the association between fractures after MM diagnosis and mortality using the Korean National Health Insurance Service database. Fracture incidence was compared between MM patients (n = 9365) and 1:1 matched control group from general population. MM patients demonstrated a significantly higher cumulative incidence of fractures, and vertebral and hip fractures presented a particularly elevated hazard ratio (1.36 [95% CI 1.18–1.55] and 1.47 [95% CI 1.10–1.97], respectively). Furthermore, the presence of fracture within the first year of MM diagnosis were associated with increased mortality (any fracture—HR 1.37 [95% CI 1.19–1.58]; vertebral fractures—HR 1.39 [95% CI 1.19–1.63]; hip fractures—HR 2.46 [95% CI 1.52–3.99]; upper limb fractures—HR 1.94 [95% CI 1.32–2.87]). These results showed an increased risk of fracture and a correlation between fractures and increased mortality in MM patients, with hip fractures notably doubling the mortality risk. These findings underscore the importance of monitoring and managing bone health in MM patients to improve survival outcomes.

## Introduction

Multiple myeloma (MM) is a hematologic malignancy characterized by uncontrolled proliferation of plasma cells^1,2^. Although its overall incidence is relatively low, it is the second most common hematologic malignancy^3^. As of 2018, South Korea reported an age-standardized incidence rate for MM at 1.7 per 100,000. This statistic has shown a consistent increase, suggesting growing prevalence of the disease within the population^4,5^. Clinically, MM manifests with specific symptoms including osteolytic lesions, renal abnormalities, elevated blood calcium levels, and anemia^2,6^. Remarkably, up to 80% of patients newly diagnosed with MM present with osteolytic lesions^7,8^. These complications often lead to bone-related problems, particularly MM-associated bone diseases. Affected patients encounter a range of skeletal problems, spanning from pathological fractures to requiring surgical intervention or radiation therapy^9,10^. These skeletal issues not only exacerbate the severity of the disease but also impose a substantial socio-economic burden on public health^9^.

An essential aspect of MM progression is the high prevalence of skeletal complications, with pathological fractures being of particular concern^11^. The increased susceptibility to fractures in MM patients results from disruption of bone homeostasis caused by myeloma cell infiltration^12^. Beyond physical consequences, these fractures significantly diminish the patient’s quality of life and limit mobility^13–15^. Furthermore, fractures, especially in critical areas such as the spine and hip, pose an elevated mortality risk, consequently reducing life expectancy of individuals with MM^6,14^.

Although the link between MM and bone diseases is well established, there is a significant void in extensive population-based research exploring the connection between fractures and mortality in MM patients. The precise impact of fractures on survival rates, the varying effects of specific fracture types (such as vertebral, hip, and upper limb fractures) on mortality, and the potential benefits of fracture management for survival outcomes remain uncertain. Only a limited number of recent population-based studies, particularly those involving large cohorts, have provided a comprehensive perspective on the interplay between fractures and mortality in individuals diagnosed with MM^16,17^.

To address these critical questions, we conducted an extensive retrospective cohort study using data from the Korean National Health Insurance Service (KNHIS) database. Our primary objectives encompassed determination of fracture incidence in MM patients in comparison to that of the general population, gaining insights into the relationship between fractures and mortality in MM and discerning the distinct impacts of various fracture types on patient longevity. Through this endeavor, we aimed to untangle the intricate interplay between fractures and MM-related mortality.

## Results

### Baseline characteristics of study population

A total of 9367 patients in the case cohort and 90,416 individuals in the control group were identified before propensity score matching. Prior to the final propensity score matching, there were noticeable differences in baseline characteristics between the cohorts, in terms of age, sex, and comorbidities (Table 1). After 1:1 propensity score matching, which was adjusted for factors including age, sex, index year, socioeconomic status, and comorbidities, the final case and control cohorts (n = 9365 each) were defined. No significant differences were observed in sex, comorbidities, or socioeconomic status between the two cohorts. All SMDs for the variables were less than 0.1, and the scores were balanced between the two cohorts, emphasizing the achieved balance in the baseline characteristics (Supplementary Fig. 1)^18^.

**Table 1:** Baseline characteristics of study population before and after propensity score matching.

	Before propensity score matching			After propensity score matching ^a^		
	Case cohort (n = 9367)	Control cohort (n = 90,416)	SMD	Case cohort (n = 9365)	Control cohort (n = 9365)	SMD
Follow-up time^b^ (years)	5.4 (5.2–5.5)	11.2 (11.2–11.2)		5.4 (5.2–5.5)	11.3 (11.2–11.4)	
Age (years)	64.5 ± 11.4	65.2 ± 11.5	0.07	64.5 ± 11.4	64.5 ± 11.6	< 0.01
Sex			0.06			< 0.01
Female	4001 (42.7)	36,075 (39.9)		4000 (42.7)	4006 (42.8)	
Male	5366 (57.3)	54,341 (60.1)		5365 (57.3)	5359 (57.2)	
Socioeconomic status			0.03			< 0.01
Low	1627 (17.4)	14,649 (16.2)		1626 (17.4)	1649 (17.6)	
Middle-high	7740 (82.6)	75,767 (83.8)		7739 (82.6)	7716 (82.4)	
Comorbidity						
MI	125 (1.3)	1107 (1.2)	0.01	125 (1.3)	132 (1.4)	< 0.01
CHF	405 (4.3)	2937 (3.2)	0.06	405 (4.3)	400 (4.3)	< 0.01
PVD	593 (6.3)	4920 (5.4)	0.04	593 (6.3)	587 (6.3)	< 0.01
CVD	1067 (11.4)	9550 (10.6)	0.03	1065 (11.4)	1070 (11.4)	< 0.01
Dementia	224 (2.4)	2432 (2.7)	0.02	224 (2.4)	193 (2.1)	0.02
Hemiplegia or paraplegia	51 (0.5)	641 (0.7)	0.02	51 (0.5)	44 (0.5)	0.01
Autoimmune disease	443 (4.7)	3017 (3.3)	0.07	442 (4.7)	432 (4.6)	< 0.01
CPD	3073 (32.8)	26,994 (29.9)	0.06	3071 (32.8)	3058 (32.7)	< 0.01
Peptic ulcer disease	2072 (22.1)	18,758 (20.7)	0.03	2071 (22.1)	2037 (21.8)	< 0.01
Hepatic disease	1205 (12.9)	10,178 (11.3)	0.05	1205 (12.9)	1168 (12.5)	0.01
Renal disease	356 (3.8)	1152 (1.3)	0.16	354 (3.8)	353 (3.8)	< 0.01
Diabetes	1807 (19.3)	15,815 (17.5)	0.04	1806 (19.3)	1827 (19.5)	< 0.01
Any cancer	826 (8.8)	6316 (7.0)	0.07	824 (8.8)	806 (8.6)	< 0.01
AIDS/HIV	2 (0.0)	12 (0.0)	< 0.01	2 (0.0)	4 (0.0)	0.01

Continuous variables are presented as mean ± standard deviation(SD); categorical variables are presented as n (%); a, the propensity score model included age, sex, index year, socioeconomic status, and prior diseases conditions; b, follow-up time represented as median follow-up (95% CI) using the reverse Kaplan–Meier estimator.

*SMD* standardized mean difference, *MI* myocardial infarction, *CHF* congestive heart failure, *PVD* peripheral vascular disease, *CVD* cerebrovascular disease, *CPD* chronic pulmonary disease.

### Comparison of cumulative incidence of fractures between MM patients and control cohort

**Fig. 1:**
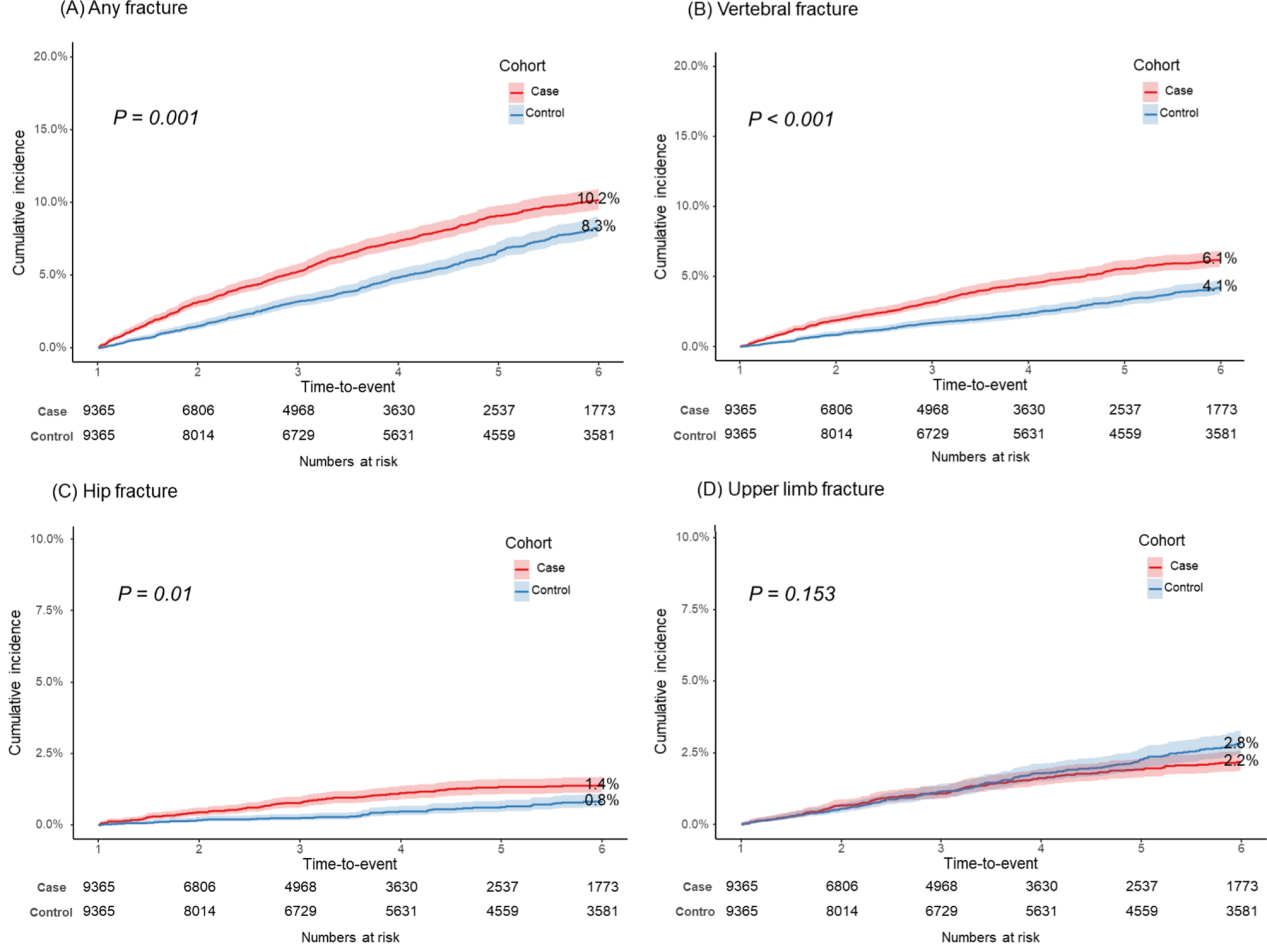
Six-year cumulative incidence curves of fractures in case and control groups after using the 1-year landmark time point by fracture subtypes. (**A**) Any fracture, (**B**) Vertebral fracture, (**C**) Hip fracture, and (**D**) Upper limb fracture. Shaded areas represent 95% CI. P shows the p-value of Gray’s test.

**Fig. 2:**
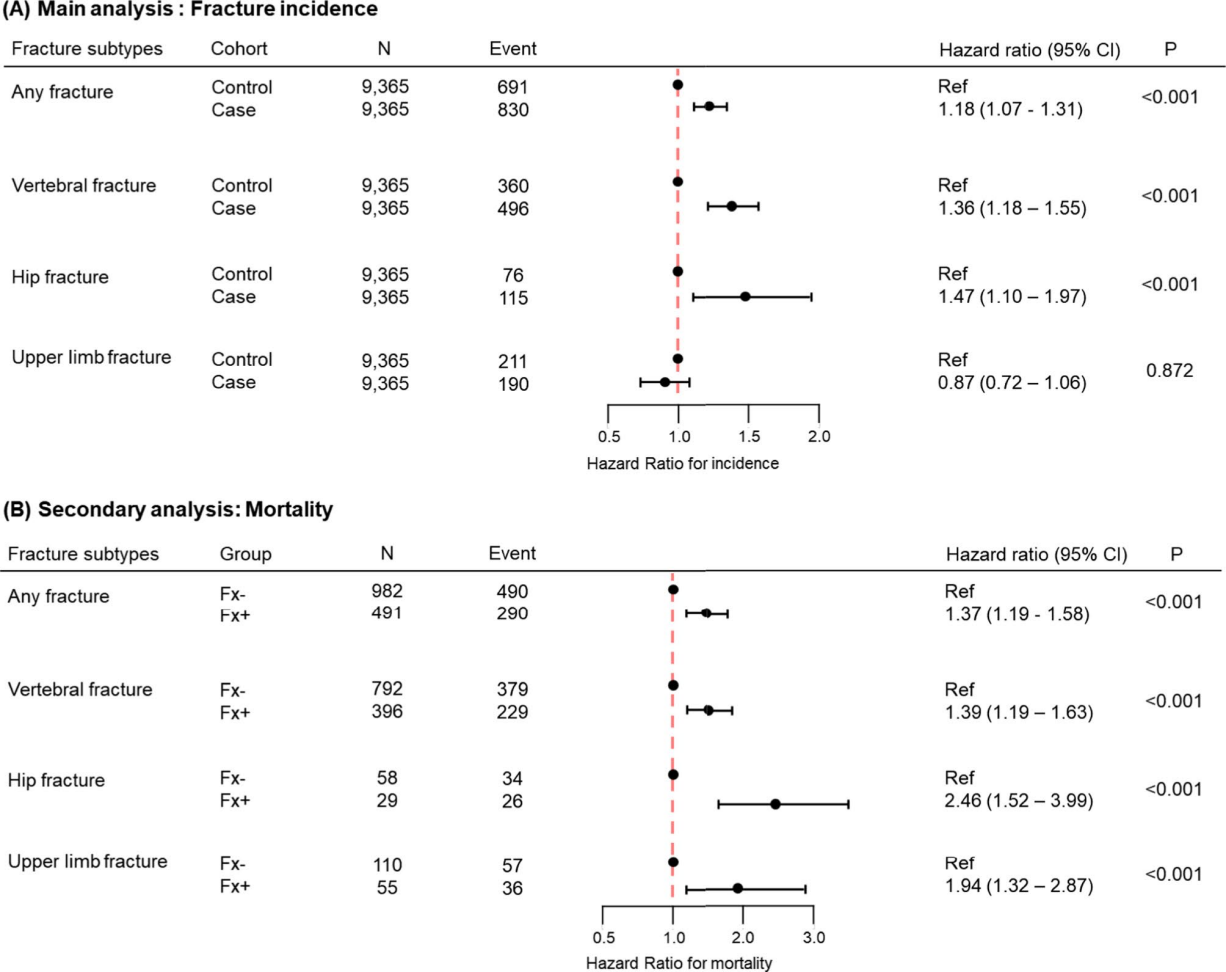
Hazard ratios for (**A**) fracture incidence among MM patients compared with healthy matched controls and (**B**) mortality among MM patients with fractures compared with those without fractures.

Figure 1 illustrates the cumulative incidence of fractures in the case and control cohorts. The 6-year cumulative incidence of any fracture was 10.2% (95% CI 9.4–11.0) in the case cohort, whereas it was 8.3% (95% CI 7.5–9.1) in the control cohort (p < 0.001, Fig. 1A). The 6-year cumulative incidence of vertebral and hip fracture in the case cohort was significantly higher than that in the control cohort: 6.1% (95% CI 5.5–6.7) vs. 4.1% (95% CI 3.5–4.7) (p < 0.001, Fig. 1B) for vertebral fractures and 1.4% (95% CI 1.2–1.6) vs. 0.8% (95% CI 0.6–1.0) (p < 0.001, Fig. 1C), for hip fractures. In terms of the cumulative incidence of upper limb fractures, there was no statistically significant difference between case (2.2%, 95% CI 1.8–2.6) and control (2.8%, 95% CI 2.4–3.2) (p = 0.153, Fig. 1D) cohorts. For any fracture, and vertebral and hip fractures, MM patients consistently showed a significantly elevated risk compared with controls at 2, 4, and 6-years after the index date (Table 2). In the regression analysis, the case cohort had significant risks for vertebral and hip fractures. HRs were as follows: any fracture–1.18 (95% CI 1.07–1.31), vertebral fractures–1.36 (95% CI 1.18–1.55), hip fractures- 1.47 (95% CI 1.10–1.97), compared with the control cohort (Fig. 2A).

**Table 2:** Cumulative incidence rates of fracture types in case and control groups by time points.

Type of fracture	Cumulative incidence rates (95% CI)			P
	At 2 years	At 4 years	At 6 years	
Any fracture				0.001
Case	3.1 (2.7–3.5)	7.3 (6.7–7.9)	10.2 (9.4–11.0)	
Control	1.5 (1.3–1.7)	4.9 (4.5–5.3)	8.3 (7.5–9.1)	
Vertebral fracture				< 0.001
Case	1.9 (1.7–2.1)	4.4 (4.0–4.8)	6.1 (5.5–6.7)	
Control	0.8 (0.6–1.0)	2.3 (1.9–2.7)	4.1 (3.5–4.7)	
Hip fracture				0.01
Case	0.4 (0.2–0.6)	1.1 (0.9–1.3)	1.4 (1.2–1.6)	
Control	0.1 (0.1–0.1)	0.5 (0.3–0.7)	0.8 (0.6–1.0)	
Upper limb fracture				0.153
Case	0.6 (0.4–0.8)	1.6 (1.4–1.8)	2.2 (1.8–2.6)	
Control	0.5 (0.3–0.7)	1.6 (1.4–1.8)	2.8 (2.4–3.2)	

*P* P-value; Cumulative incidence rates are presented as % (95% CI); the p-value represents the Gray’s test result.

### Secondary analysis on mortality in MM patients based on fracture diagnosis

**Fig. 3:**
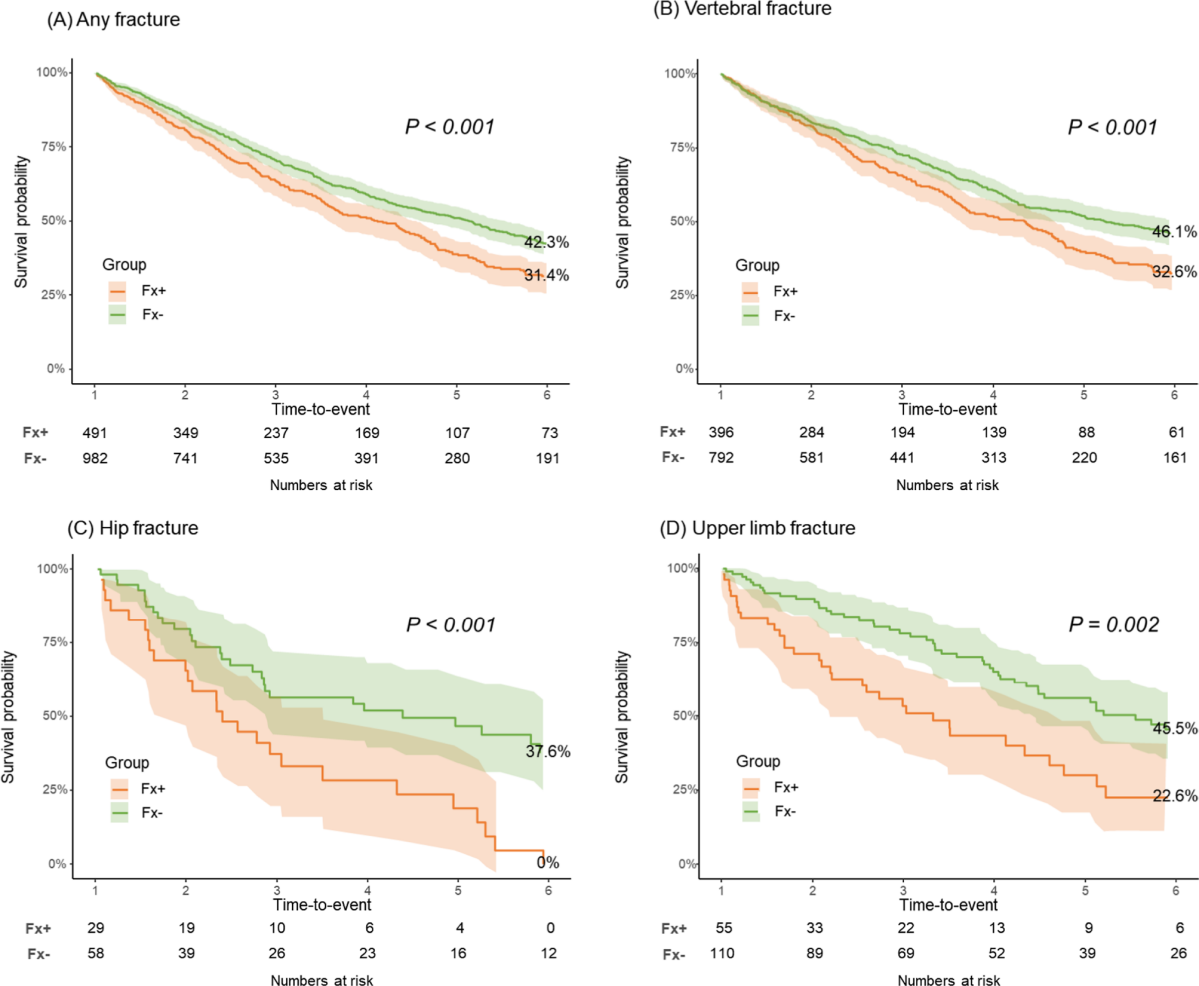
Kaplan-–Meier estimates of overall survival in MM patients with and without fractures. (**A**) Any fracture. (**B**) Vertebral fracture. (**C**) Hip fracture. (**D**) Upper limb fracture. Shaded areas represent 95% CI. P shows the p-value of the log-rank test.

A secondary analysis was conducted to examine the impact of early fractures within 1 year of MM diagnosis on mortality. For this analysis, 491 individuals who experienced a predefined fracture within 1 year of MM diagnosis were identified in the final case cohort, and this group was referred to as the Fx+ group. The Fx- group was developed by matching the Fx+ group at a 1:2 ratio through a propensity score among MM patients who had not experienced a predefined fracture event within 1 year from MM diagnosis (Supplementary Fig. 2)^18^. Both pre- and post-matching characteristics were assessed, ensuring a balanced comparison, as evidenced by the SMDs being below 0.1 (Supplementary Table 1)^18^. For any fracture, the Fx+ group had a higher risk of death than the Fx- group, with an HR of 1.37 (95% CI 1.19–1.58) with a lower survival probability (Fig. 2B, Fig. 3A). Considering predefined fracture subtypes, the HRs of the Fx+ group with all major subtypes were found to be significantly elevated (vertebral, 1.39 [95% CI 1.19–1.63, Figs. 2B, 3B]; hip, 2.46 [95% CI 1.52–3.99, Figs. 2B, 3C]; upper limb, 1.94 [95% CI 1.32–2.87, Figs. 2B, 3D]) with a low survival probability when compared with those of matched individuals in the Fx- group. The 6-year survival rate was lower in those with any fracture (16.9% vs. 30.7%, 95% CI 12.5–22.7 vs 26.8–35.1, p < 0.001) and those with other fracture subtypes showed consistent results compared with MM patients without such fractures (Table 3).

**Table 3:** Survival rates of multiple myeloma patients with and without fractures by time points.

Type of fracture	Survival rates (95% CI)			P
	At 2 years	At 4 years	At 6 years	
Any fracture				< 0.001
Fx +	80.9 (77.3–84.5)	51.1 (46.3–56.3)	31.4 (26.6–36.9)	
Fx-	85.2 (82.9–87.5)	59.0 (55.7–62.6)	42.3 (38.7–46.4)	
Vertebral fracture				< 0.001
Fx +	82.5 (78.8–86.5)	51.7 (46.4–57.6)	32.6 (27.3–38.9)	
Fx-	83.7 (81.1–86.4)	60.8 (57.1–64.8)	46.1 (42.0–50.6)	
Hip fracture				< 0.001
Fx +	65.5 (50.3–85.3)	28.5 (15.5–52.4)	19.0 (8.3–43.6)*	
Fx-	79.6 (69.5–91.1)	52.0 (39.7–68.2)	37.6 (25.3–55.9)	
Upper limb fracture				0.002
Fx +	71.3 (60.0–84.8)	43.5 (31.1–60.7)	22.6 (12.0–42.6)	
Fx-	89.7 (84.2–95.7)	65.2 (56.1–75.7)	45.5 (35.6–58.0)	

*P* P-value; Survival rates are presented as % (95% CI); The p-value represents the Log rank test result; *Survival rates at 5 years.

## Discussion

In our analysis of population-based case-cohort data of 13,925 MM patients from the KNHIS database, we assessed the prevalence of fractures and their potential association with mortality. Our findings revealed a significantly higher incidence of vertebral and hip fractures in MM patients (case cohort) than in the general population without MM (control cohort). Moreover, among MM patients in the case cohort, those who experienced fractures within 1 year of MM diagnosis demonstrated a significantly higher mortality rate, regardless of the fracture type. In particular, MM patients with hip fractures exhibited a significant increase in mortality compared with MM patients without such fractures.

In our study, the cumulative risk of fractures was found to be consistently higher in MM patients than in the general population. These findings are consistent with those of previous population-based studies^16,17^. Previous studies investigating fractures in MM patients have reported similar trends, albeit in different populations and using different analytical methods. Melton et al. analyzed 165 MM patients and observed a modest increase in the risk of osteoporotic fractures, particularly in the hip, spine, and wrist^16^. The standardized incidence ratio (SIR) for fractures combined was reported as 2.1 (95% CI 1.2–3.5)^16^. While this study highlighted an increase in the incidence of fractures following MM diagnosis, it did not include a control group for a direct comparison, cumulative fracture risk tracking over time, or detailed results according to specific fracture sites, making direct comparisons with our study challenging. Recently, Thorsteinsdottir et al. conducted a population-based study on fractures and survival in MM patients, using a cohort from Sweden^17^. Their study included a substantial cohort of 14,013 individuals and identified an increased risk of fractures following MM diagnosis. This rigorous analysis of a large cohort of MM patients is similar in scale to ours, and their contributions are invaluable because of the comprehensive assessment of fracture risk in the MM population.

Factors contributing to the increased susceptibility of MM patients to fractures have been extensively studied^1,19^. MM is characterized by plasma cell dyscrasia, leading to the frequent development of osteolytic lesions, which in turn increases the risk of fractures^7^. Bone remodeling is regulated by interactions between various bone microenvironments^20^. However, in MM, these interactions are disrupted, leading to increased bone loss owing to elevated osteoclast activity and reduced osteoblast function^7,21^. Osteocytes, which are essential for maintaining bone homeostasis and are known to release molecules such as the NF-κB ligand receptor activator, sclerostin, and Dickkopf-1^22–26^ are induced by MM cells to undergo apoptosis. This alters the bone marrow microenvironment and creates a metastatic niche for MM cells^27^. As a result, MM patients have a reduced number of functional osteoblasts, compromised bone quality, and increased fracture risk^28,29^.

A distinctive aspect of our study was the analysis of mortality based on fracture status, particularly within the cohort of MM patients. Our research revealed that MM patients who experienced a fracture within 1 year of MM diagnosis had a higher mortality rate than those without a fracture within 1 year of MM diagnosis. Importantly, the HR for mortality significantly increased for all types of fractures. The association between the increased incidence of fractures and mortality, irrespective of specific conditions, such as MM, is well-established^30–33^. In osteoporosis patients, the mortality rate after hip fractures is typically higher than that after vertebral fractures^34,35^. In our analysis, MM patients with hip fractures that occurred within 1 year of MM diagnosis demonstrated the highest mortality (HR 2.46, 95% CI 1.52–3.99). Hip fractures can lead to functional limitations and often necessitate prolonged bed rest. During this period, patients are susceptible to pressure ulcers, infections, and pneumonia, which may increase the risk of mortality^36–38^. MM patients are likely to develop various fracture sequelae as well as postoperative complications after a hip fracture, leading to an overall increase in mortality.

Our findings have significant clinical implications and highlight the intricate relationships among fractures, mortality, and MM. This emphasizes the importance of fracture prevention and management in these patients. Given the elevated mortality risk associated with fractures in MM, healthcare professionals should prioritize routine bone health assessments and consider interventions to reduce fracture risk. MM patients can experience not only pathological fractures but also low-energy fractures due to disruption of bone homeostasis and reduced bone strength^39^. Existing clinical guidelines emphasize the use of bone-modifying agents such as bisphosphonates and denosumab to address MM-related bone complications^14,40–44^. MM patients who experienced a fracture within 1 year of diagnosis had a significantly increased mortality rate compared with those who did not experience a fracture within 1 year of diagnosis; therefore, the risk of fracture should be minimized after MM diagnosis. Although there are concerns about the long-term side effects of antiresorptive agents, the benefits of treatment far outweigh the adverse effects in these patients, and efforts to prevent fractures, including active medication and education, are necessary.

The present study has some limitations. First, our assessment of the fracture incidence relied on hospital records rather than definitive diagnoses. Although the Korean public insurance system extensively employs ICD-10 codes from medical records that undergo rigorous monitoring, particularly for conditions such as hematologic malignancies and fractures, variations may still exist. However, these codes, subject to a meticulous review owing to insurance coverage discrepancies, likely reflect the prevailing clinical practices in Korea. Second, our dataset lacked information on critical clinical details of MM patients, such as their remission status, medication types, nutritional health, physical activity habits, and other pertinent MM-related characteristics. In particular, the use of bone-modifying agents could not be verified within this dataset. However, according to the standard of care for MM, patients diagnosed with MM receive bisphosphonates. Despite this, our findings highlight a significantly elevated fracture risk in MM patients, which underscores important clinical implications. Furthermore, this analysis employs ICD-10 diagnosis codes, posing a challenge in differentiating between low-energy fractures and pathologic fractures. Although our study provides an overview of the fracture incidence and mortality in real-world clinical settings, a more in-depth examination of these factors would yield a better and more nuanced understanding. Furthermore, there are limitations to the availability of specific data. The absence of information on the clinical stage, prognostic factors, and administered treatments precluded us from definitively stating whether fractures act as independent risk factors for mortality in MM. Notably, as the fractures occurred after MM diagnosis, there was a potential bias in our fracture incidence and following survival analysis. To mitigate this, we incorporated landmark analysis to accurately compare survival outcomes between patients with and without fractures. Third, the lack of access to radiographic evidence could have led to underreporting, particularly for asymptomatic fractures. However, it is also possible that in patients with MM, more frequent imaging is performed, leading to a higher detection rate of asymptomatic fractures. As our study focused exclusively on Korean patients with MM, generalizing the findings to a broader population may be challenging.

To the best of our knowledge, this is the first comprehensive national case–control study in Korea to identify the incidence of fractures in patients with MM by site and to examine the association of fractures with mortality. Our findings confirm that MM patients are at an increased risk of fractures compared with the general population. In addition, patients who experienced a fracture within 1 year of their MM diagnosis had a significant increase in mortality compared with those who did not. In particular, femoral fractures have the worst prognosis among MM patients. Considering this vulnerability, clinicians must actively monitor fracture risk, administer appropriate bone-modifying treatments, and offer thorough education on fracture prevention in MM patients.

## Methods

### Data source

This study used data from the KNHIS database, a comprehensive healthcare insurance system that provides healthcare coverage for all South Korean individuals. The database encompasses health-related information of approximately 50 million individuals and incorporates sociodemographic data, medical diagnoses based on the International Statistical Classification of Diseases, Tenth Revision, Clinical Modification (ICD-10-CM), and treatment data for the Korean population^45^. This study was approved by the Institutional Review Board of Seoul St. Mary’s Hospital in Seoul, Korea (approval number: KC21ZNSI0448) and strictly adhered to the principles articulated in the Declaration of Helsinki, in addition to other relevant regulations and guidelines. As this study involved the analysis of anonymized and de-identified publicly accessible data, the need for obtaining informed consent was waived.

### Study population and design

This study had a retrospective cohort design. The initial case cohort, referred to as the “primitive case cohort,” consisted of 37,883 eligible patients diagnosed with MM (ICD-10-CM code: C90) between January 2009 and December 2020. Simultaneously, an initial control cohort was established from the population covered by the KNHIS database, including individuals without an MM diagnosis code. Subsequently, 378,830 individuals were enrolled in the primitive control cohort by selection using a 1:10 propensity matching method based on the birth year and sex of the primitive case cohort.

An intermediate-case cohort (n = 13,925) was derived from the primitive-case cohort based on defined inclusion and exclusion criteria. Initially, 24,451 patients with a principal diagnosis code for MM (C90) were selected based on the inclusion criteria. This approach was chosen because individuals for whom the MM diagnosis was solely based on non-principal diagnostic codes were considered challenging to definitively confirm as having MM. The following exclusion criteria were applied: 1) missing follow-up data (n = 49), 2) insufficient socioeconomic status data (n = 332), 3) age under 19 years (n = 52), 4) MM diagnosis in 2009 (n = 3,588) to ensure a minimum one-year claim data, 5) insufficient MM diagnosis due to the MM code appearing only once (n = 2,808), and 6) a history of fracture before the diagnosis of MM (n = 3,697) to avoid potential bias from pre-existing fractures affecting the event of fracture following MM diagnosis. The intermediate control cohort (n = 111,400) was created through a 1:8 propensity score-matching process based on the birth year and sex of the intermediate case cohort. This matching aimed to establish an index date for the final control cohort. The index date for individuals in the intermediate-control cohort was set as the date of MM diagnosis in the intermediate-case cohort.

**Fig. 4:**
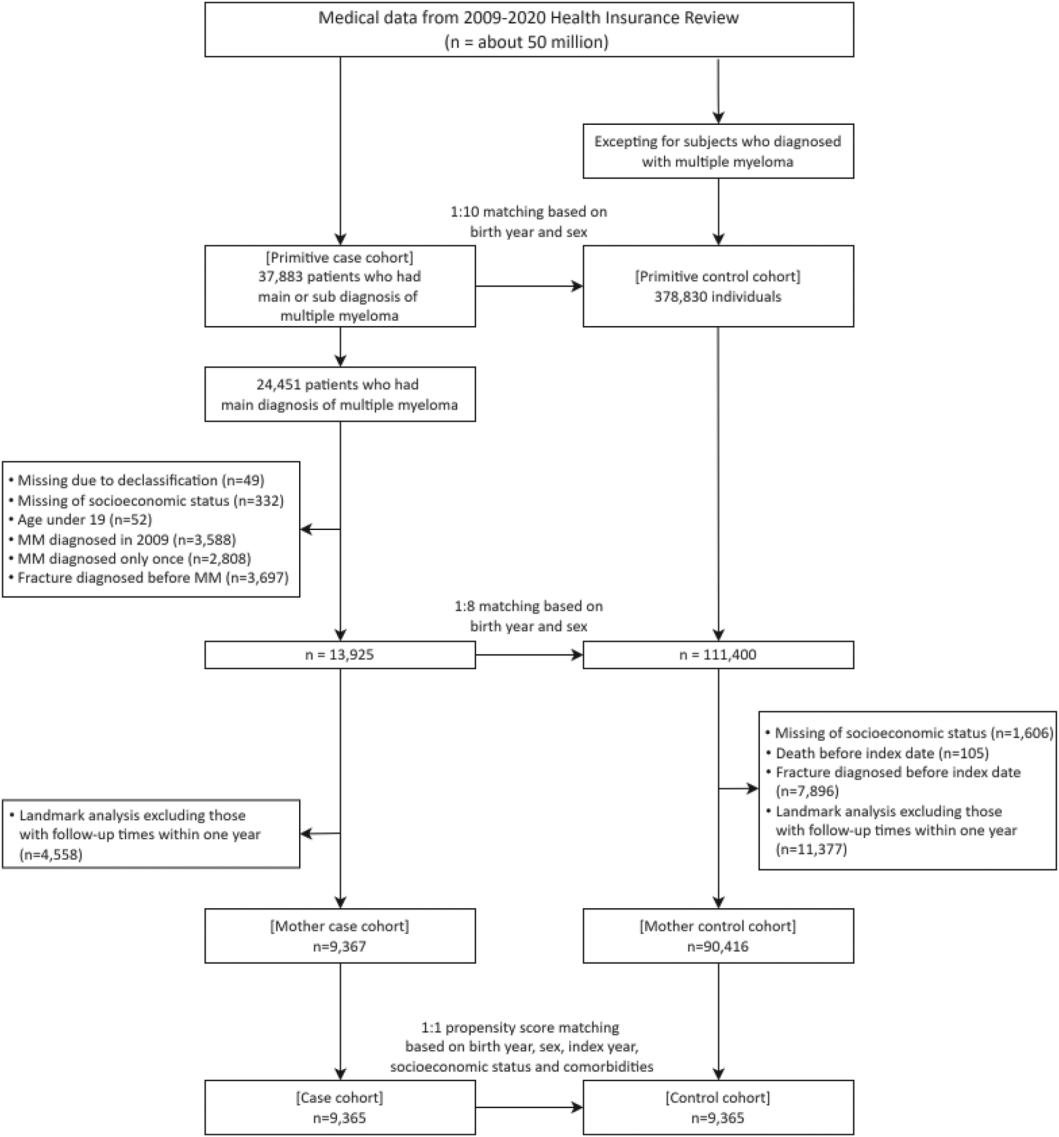
Flowchart of the study population selection process. Landmark analysis excluded patients with death, loss to follow-up, or any fracture within 1 year from diagnosis.

Within the intermediate-control cohort, patients with an event of death or fracture before the index date (105 and 7,896 cases, respectively) or missing socioeconomic status data (n = 1,606) were further excluded. To facilitate a 1-year landmark analysis in both the case and control cohorts, individuals with a follow-up time within 1 year from the index date were excluded, resulting in the exclusion of 4,558 and 11,377 cases from each cohort, respectively. The final case and control cohorts (n = 9,365 each) were established through a 1:1 propensity score matching process based on the birth year, sex, index year, socioeconomic status, and comorbidities on the index date according to the Charlson Comorbidity Index (CCI)^46^. These comorbidities included previous myocardial infarction, congestive heart failure, peripheral vascular disease, cerebrovascular disease, dementia, hemiplegia or paraplegia, autoimmune disease, chronic pulmonary disease, peptic ulcer disease, hepatic disease, renal disease, and diabetes, with or without chronic complications. The process of selecting the final case and control cohorts is illustrated in Fig. 4.

### Definition of fracture event

Fracture events were defined using specific ICD-10 codes in alignment with those recommended by the Korean Endocrine Society to ensure data consistency in the nationwide research^47^. Specifically, a vertebral fracture was characterized using the ICD-10 code M48.4, M48.5, M49.5, M80.88, S22.0, S22.1, S32.0, S32.7, or T08. A hip fracture was identified by the presence of the ICD-10 code S72.0 or S72.1, noted at least once during one incidence of hospitalization. Upper limb fractures were defined by recording the following ICD-10 code: S42.2, S42.3, S52.5, or S52.6. The category of "any fracture” encompassed any ICD-10 codes of the aforementioned fracture subtypes and fractures specified as “other fracture” (ICD-10 code S42.0, S82.3, S82.5, or S82.6). These fractures were considered if they appeared as either a principal or auxiliary diagnosis in the medical expense claims submitted to the KNHIS, until the end of the follow-up period (December 31, 2020). For fractures recorded as an auxiliary diagnosis, the occurrence of two diagnoses was considered an event, with the event time defined as the date of the second diagnosis. Fracture events were excluded if the fracture was associated with a trauma code (V01-99, X00-58, X59.9, W20-99, T00-01, T03-07, W02-04, W09, or W11-17).

### Covariates

For baseline characteristics, data were collected from records at the index date. The CCI was used to assess comorbidities prior to the diagnosis of MM, based on medical records from at least six months before the index date. The CCI was then calculated using the comorbidities identified up to the time point of six months prior to the index date^46^. A 6-month washout period was used to minimize bias due to potential symptoms or signs related to MM. Each comorbidity was defined using ICD-10-CM codes (Supplementary Table 1). To define comorbidities before MM diagnosis, a primary diagnosis or at least two entries for secondary diagnoses before the index date were required. Socioeconomic status was represented by the KNHIS, using a numeric value derived from the average monthly insurance premium; 0 was encoded for medical aid and 1–20 for evenly distributed percentiles^48^. These groups were further categorized into two main categories: the lower 1st to 3rd percentiles (scores 0, 1, and 2) and the 4^th^ to 21^st^ percentiles (scores 3 to 20).

### Propensity score matching

Propensity score matching was employed to account for variations in baseline characteristics between the case and control cohorts. The propensity score was computed using a logistic regression model, considering factors such as age, sex, index year, socioeconomic status, and CCI-based comorbidities with a prevalence of 0.1% or higher, which were included in the conditioning variables. To obtain the final study population, 1:1 propensity score matching was performed without replacement using the greedy-matching algorithm with a caliper width of 0.25. The standardized mean differences (SMDs) of each covariate across cohorts before and after propensity score matching were calculated and assessed as balanced when the SMD was less than 0.1^49^.

### Statistical analyses

Continuous variables were reported as means with their corresponding standard deviations, while categorical variables were presented as frequencies or percentages. Analyses of normally distributed continuous variables were performed by conducting two-sample t-tests, whereas non-normally distributed continuous variables were assessed using the Wilcoxon rank-sum tests. Chi-squared tests were used for categorical variables, and Fisher’s exact test was used when the expected frequency was < 5. Gray’s test was employed to assess differences in the cumulative incidence of fractures, while accounting for death as a competing risk factor. Fine–Gray regression model was generated with hazard ratios (HRs) and 95% confidence intervals (CIs) for multivariate analyses. To account for the clustering of matched pairs, statistical inference was based on the Fine–Gray proportional hazard model with robust standard errors using sandwich covariance matrix estimation. A landmark analysis method was employed to minimize the potential for immortal time bias. This method excluded patients who experienced an event (Primary analysis: death, loss to follow-up, or fracture event; Secondary analysis: death or loss to follow-up) between the index date and a predetermined landmark time point. For fractures recorded as an auxiliary diagnosis, the second diagnosis within 1-year was excluded. The landmark time was set at 1 year, thus excluding individuals with a follow-up time of less than 1 year. All statistical tests were two-tailed, and a P-value of < 0.05 was considered to indicate statistical significance. Analyses were conducted using SAS version 9.4 (SAS Institute Inc.) and R version 4.0.3 (R Foundation for Statistical Computing, Vienna, Austria).

### Secondary analysis

Additional analyses were performed to compare the risk of mortality between two subgroups: MM patients diagnosed with fractures within 1 year following their MM diagnosis (Fx+ group) and those who were not (Fx- group) (Supplementary Fig. 2)^18^. MM patients with any fracture within 1 year were defined as those who received the specified ICD-10 code as the principal diagnosis at least once or as an auxiliary diagnosis at least twice. For auxiliary diagnosis, if the first recorded diagnosis occurred within 1 year, the patient was classified as part of the Fx+ group. The risk of mortality among different fracture subtypes was compared by defining the subtypes using the same ICD-10 codes, requiring at least one principal diagnosis or at least two secondary diagnoses. Each fracture subtype comprised an independent cohort. The Fx- group was selected based on 1:2 propensity score matching to ensure comparability with precision. Survival probabilities were determined using the Kaplan–Meier method for both the Fx+ and Fx- groups. After obtaining these survival curves, the log-rank test was used to identify significant disparities in survival probabilities between the groups. To further enhance our analysis, the Cox proportional hazards model was used to estimate the HR and its 95% CI using the sandwich covariance matrix to account for the clustering of matched pairs.

## Supplementary Information

Supplementary Information 1.

Supplementary Information 2.

Supplementary Information 3.

## Data Availability

Some or all datasets generated and/or analyzed during the current study are not publicly available but can be obtained from the corresponding author upon reasonable request. However, certain datasets have a data usage expiration date.
