# Supplementary material for: Fractures incidence and its association on mortality in multiple myeloma patients: a nationwide cohort study (CAREMM-2105 study)
Source: Sci Rep. 2025 Jul 27;15:27321. doi: 10.1038/s41598-025-09811-4 (PMC12301462; doi:10.1038/s41598-025-09811-4)
Supplement: Supplementary file 1 — Supplementary Information 1. [file 41598_2025_9811_MOESM1_ESM.pptx]

## Slide 1
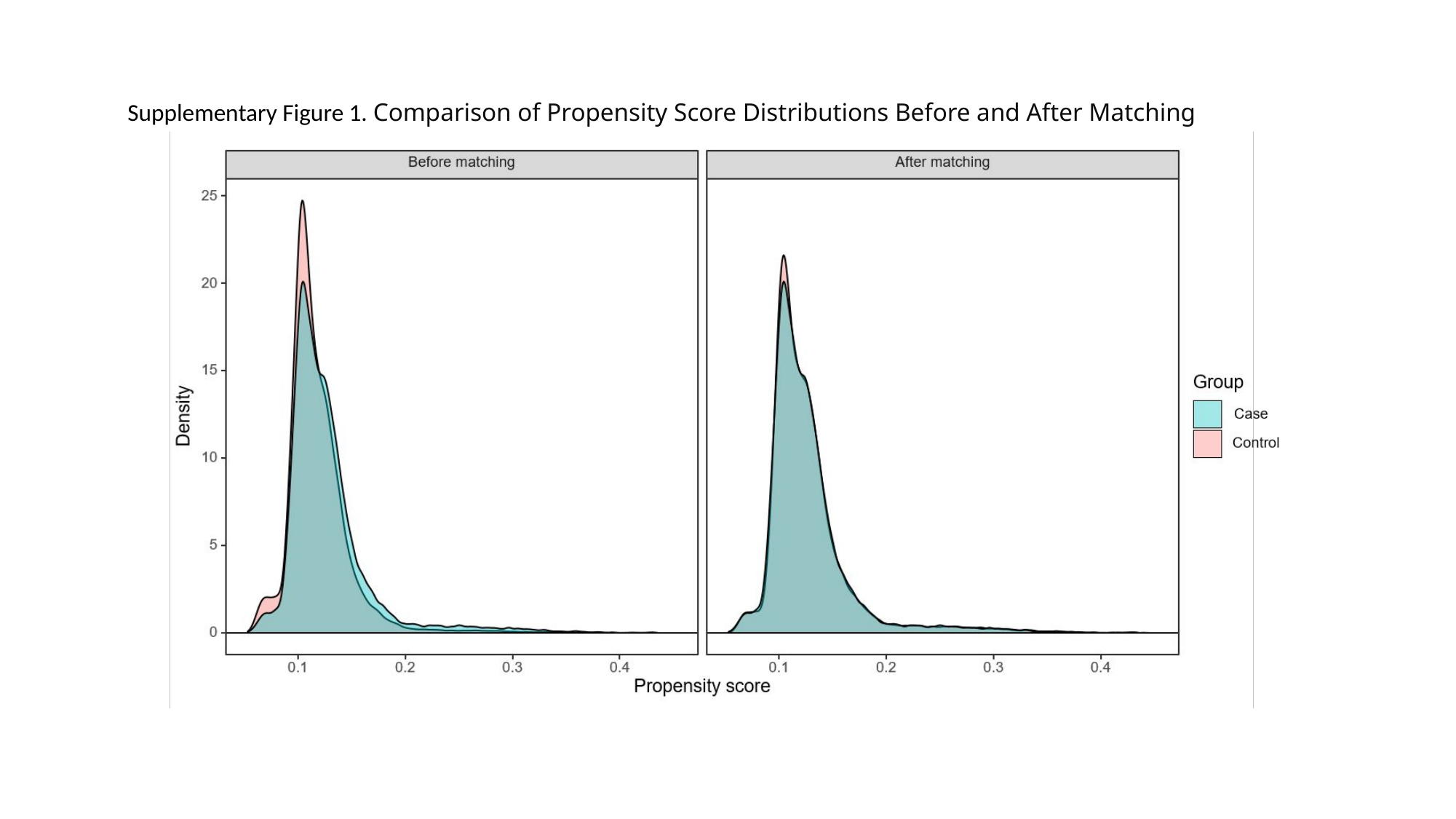

Supplementary Figure 1. Comparison of Propensity Score Distributions Before and After Matching
