# Supplementary material for: Fractures incidence and its association on mortality in multiple myeloma patients: a nationwide cohort study (CAREMM-2105 study)
Source: Sci Rep. 2025 Jul 27;15:27321. doi: 10.1038/s41598-025-09811-4 (PMC12301462; doi:10.1038/s41598-025-09811-4)
Supplement: Supplementary file 2 — Supplementary Information 2. [file 41598_2025_9811_MOESM2_ESM.pptx]

## Slide 1
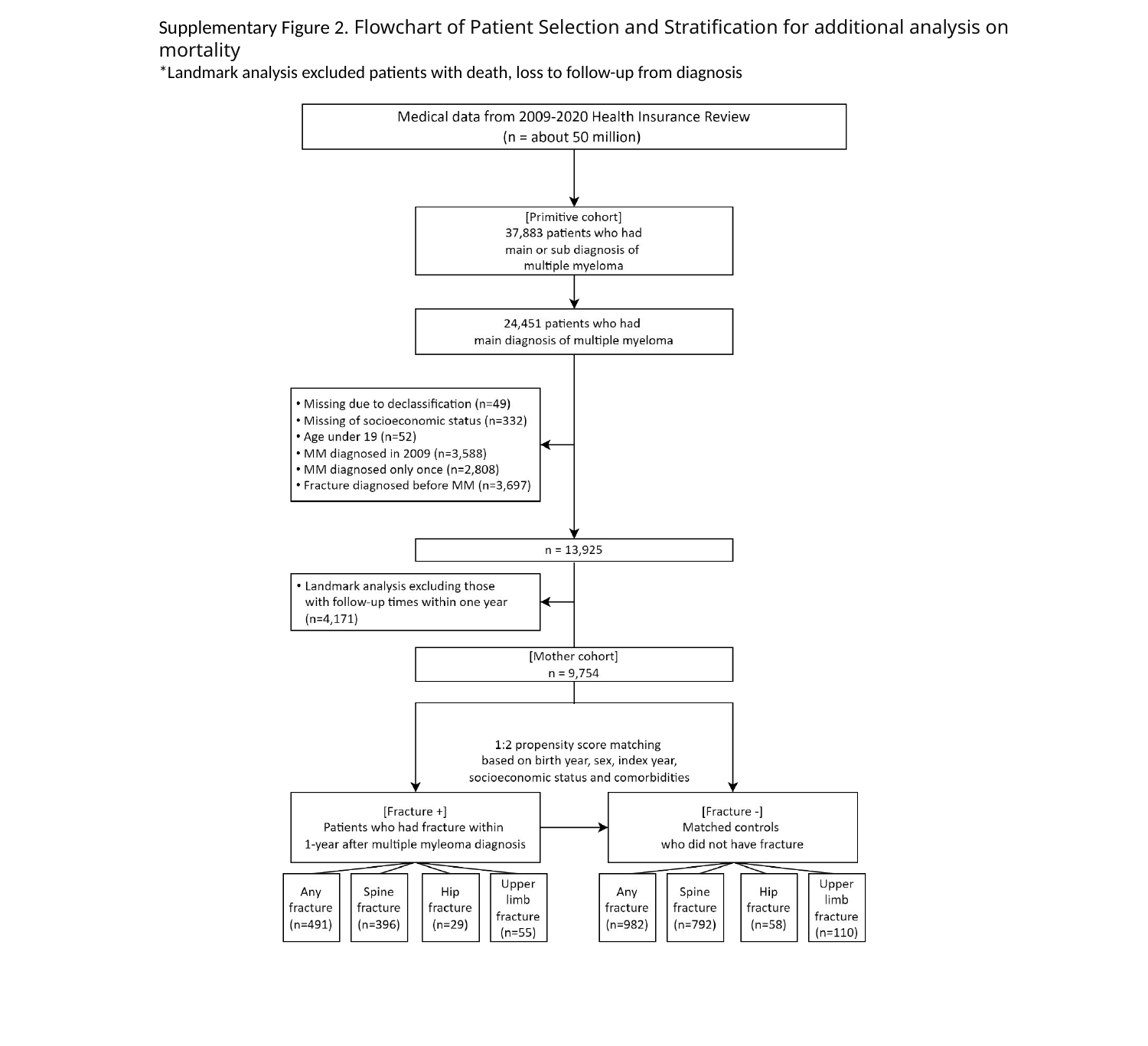

Supplementary Figure 2. Flowchart of Patient Selection and Stratification for additional analysis on mortality
*Landmark analysis excluded patients with death, loss to follow-up from diagnosis
