# Supplementary material for: Fractures incidence and its association on mortality in multiple myeloma patients: a nationwide cohort study (CAREMM-2105 study)
Source: Sci Rep. 2025 Jul 27;15:27321. doi: 10.1038/s41598-025-09811-4 (PMC12301462; doi:10.1038/s41598-025-09811-4)
Supplement: Supplementary file 3 — Supplementary Information 3. [file 41598_2025_9811_MOESM3_ESM.docx]

Supplementary table 1. Baseline characteristics of sub-cohort for any fracture cohort before and after propensity score matching

|  | Before propensity score matching | | | After propensity score matching ^a^ | | |
| --- | --- | --- | --- | --- | --- | --- |
|  | Fx+  (n = 491) | Fx-  (n = 9,263) | SMD | Fx+  (n = 491) | Fx-  (n = 982) | SMD |
| **Follow-up time**^b^ **(years)** | 4.1 [3.6 – 4.6] | 5.5 [5.3 – 5.6] |  | 4.1 [3.6 – 4.6] | 5.2 [4.6 – 5.6] |  |
| **Age (years)** | 66.1 ± 11.4 | 64.4 ± 11.3 | 0.15 | 66.1 ± 11.4 | 66.4 ± 10.9 | 0.03 |
| **Sex** |  |  | 0.15 |  |  | <0.01 |
| Female | 245 (49.9%) | 3956 (42.7%) |  | 245 (49.9%) | 495 (50.4%) |  |
| Male | 246 (50.1%) | 5307 (57.3%) |  | 246 (50.1%) | 487 (49.6%) |  |
| **Socioeconomic status** |  |  | 0.09 |  |  | 0.03 |
| Low | 102 (20.8%) | 1610 (17.4%) |  | 102 (20.8%) | 191 (19.5%) |  |
| Middle-high | 389 (79.2%) | 7653 (82.6%) |  | 389 (79.2%) | 791 (80.5%) |  |
| **Comorbidity** |  |  |  |  |  |  |
| MI | 12 ( 2.4%) | 174 ( 1.9%) | 0.04 | 12 ( 2.4%) | 26 ( 2.6%) | 0.01 |
| CHF | 33 ( 6.7%) | 749 ( 8.1%) | 0.05 | 33 ( 6.7%) | 74 ( 7.5%) | 0.03 |
| PVD | 29 ( 5.9%) | 698 ( 7.5%) | 0.07 | 29 ( 5.9%) | 53 ( 5.4%) | 0.02 |
| CVD | 69 (14.1%) | 1326 (14.3%) | <0.01 | 69 (14.1%) | 133 (13.5%) | 0.02 |
| Dementia | 29 ( 5.9%) | 419 ( 4.5%) | 0.06 | 29 ( 5.9%) | 64 ( 6.5%) | 0.03 |
| Hemiplegia or  paraplegia | 5 ( 1.0%) | 105 ( 1.1%) | 0.01 | 5 ( 1.0%) | 14 ( 1.4%) | 0.04 |
| Autoimmune disease | 30 ( 6.1%) | 533 ( 5.8%) | 0.02 | 30 ( 6.1%) | 59 ( 6.0%) | <0.01 |
| CPD | 214 (43.6%) | 3703 (40.0%) | 0.07 | 214 (43.6%) | 428 (43.6%) | <0.01 |
| Peptic ulcer disease | 143 (29.1%) | 2393 (25.8%) | 0.07 | 143 (29.1%) | 290 (29.5%) | <0.01 |
| Hepatic disease | 84 (17.1%) | 1537 (16.6%) | 0.01 | 84 (17.1%) | 156 (15.9%) | 0.03 |
| Renal disease | 65 (13.2%) | 1317 (14.2%) | 0.03 | 65 (13.2%) | 122 (12.4%) | 0.02 |
| Diabetes | 101 (20.6%) | 2245 (24.2%) | 0.09 | 101 (20.6%) | 217 (22.1%) | 0.04 |
| Any cancer | 114 (23.2%) | 2247 (24.3%) | 0.02 | 114 (23.2%) | 245 (24.9%) | 0.04 |
| AIDS/HIV | 0 ( 0.0%) | 4 ( 0.0%) | 0.03 | 0 (0.0) | 0 (0.0) | <0.01 |

Abbreviations: MM, Multiple Myeloma; SMD, Standardized mean difference; MI, myocardial infarction; CHF, Congestive heart failure; PVD, Peripheral vascular disease; CVD, Cerebrovascular disease; CPD, Chronic pulmonary disease; Continuous variables are presented as mean±standard deviation(SD); categorical variables are presented as n (%); a, the propensity score model included age, sex, index year, socioeconomic status, and prior diseases conditions; b, follow-up time represented as median follow-up (95% CI) using the reverse Kaplan-Meier estimator.
